# Development and Preclinical Safety Evaluation of an Injectable β-Caryophyllene Nanoemulsion

**DOI:** 10.3390/ph19050763

**Published:** 2026-05-13

**Authors:** Ana Bárbara Souza Viana, Natálya Gabriely Lobato-Santos, Andressa Ketelem Meireles Alberto, Abrahão Victor Tavares de Lima Teixeira dos Santos, Sergio Gabriell Leite Brito, Nayara Nilcia Dias Colares, José Carlos Tavares Carvalho

**Affiliations:** 1 Postgraduate Program in Pharmaceutical Sciences, Department of Biological and Health Sciences, Federal University of Amapá (UNIFAP), Rodovia Josmar Chaves Pinto, Km 0, Macapá 68903-419, Brazil; 2 Drug Research Laboratory, Department of Biological and Health Sciences, Federal University of Amapá (UNIFAP), Rodovia Josmar Chaves Pinto, Km 0, Macapá 68903-419, Brazil; 3 Postgraduate Program in Pharmaceutical Innovation (PPGIF), Institute of Health Sciences, Federal University of Pará (UFPA), Av. Augusto Corrêa, 01, Guamá, Belém 66075-110, Brazil; 4 University Hospital, Federal University of Amapá (UNIFAP), Rodovia Josmar Chaves Pinto, Km 0, Macapá 68900-336, Brazil

**Keywords:** β-caryophyllene, nanoemulsion, preclinical toxicology, drug delivery, pharmaceutical safety

## Abstract

**Background/Objectives:** β-Caryophyllene is a plant-derived sesquiterpene with recognized therapeutic potential; however, high lipophilicity and low aqueous solubility limit its parenteral application. Nanoemulsion-based systems represent a rational strategy to address these challenges. This study aimed to develop and physicochemically characterize an injectable β-caryophyllene nanoemulsion and to evaluate its preclinical safety following intramuscular administration. **Methods:** Five nanoemulsions (NBCP1–NBCP5) were prepared by low-energy emulsification using different hydrophilic–lipophilic balance (HLB) values and characterized in terms of particle size, polydispersity index, zeta potential, and encapsulation efficiency. The optimized formulation (NBCP1) was evaluated in a 14-day subacute intramuscular toxicity study in male Wistar rats (*n* = 5 per group) at doses of 5 and 15 mg/kg. Clinical observations, food and water intake, body weight, hematological and biochemical parameters, and histopathological analyses of muscle, liver, and kidney tissues were assessed. **Results:** NBCP1 exhibited favorable physicochemical properties, including a mean particle size of 102.39 nm, PDI of 0.27, zeta potential of −27.5 mV, and encapsulation efficiency of 97.16%, and remained stable under stress conditions. Repeated intramuscular administration did not induce behavioral alterations, changes in consumption patterns, or differences in body weight between the control and treated groups. Hematological and biochemical parameters remained within physiological ranges, and histopathological analysis revealed preserved tissue architecture without inflammatory or degenerative changes. **Conclusions:** The results support the suitability of NBCP1 as a stable nanoemulsion platform for the parenteral delivery of β-caryophyllene under the evaluated conditions. These findings address the limited information available on injectable formulations of this sesquiterpene and provide a foundation for future pharmacokinetic, longer-term safety, and efficacy studies.

## 1. Introduction

Natural bioactive compounds have long constituted a cornerstone of pharmacological innovation, providing a continuous source of structurally diverse molecules with clinically relevant biological activities [1]. A substantial proportion of contemporary drugs and drug leads originates directly or indirectly from plant-derived compounds, underscoring the enduring importance of natural chemical diversity in pharmaceutical research [2]. In this context, increasing attention has been paid to bioactive molecules derived from biodiversity-rich ecosystems, where chemical complexity, ecological abundance, and historical medicinal use converge to support translational relevance. Tropical forests, particularly the Amazon biome, are among the most significant global reservoirs of medicinal plant species, many of which biosynthesize terpenes and sesquiterpenes with well-established therapeutic potential [3,4].

Among these sesquiterpenes, β-caryophyllene has emerged as a compound of growing pharmacological interest due to its wide distribution across diverse botanical sources and its presence in plants of economic, medicinal, and ecological relevance [5]. It is a major constituent of the essential oils and oleoresins of numerous species, including *Syzygium aromaticum*, *Cannabis sativa*, and notably *Copaifera* spp. (copaíba), a genus native to the Amazon region whose oleoresins have been traditionally employed for the treatment of inflammatory and painful conditions [6,7]. This extensive botanical occurrence, coupled with growing experimental evidence, positions β-caryophyllene as a representative bioactive compound derived from a globally relevant, chemically rich biodiversity context [8]. From a physicochemical standpoint, β-caryophyllene is a bicyclic sesquiterpene with pronounced lipophilicity, exhibiting a molecular weight of approximately 204.35 g/mol and a logP value close to 4.5, properties that directly influence its formulation behavior and biopharmaceutical performance [9].

Beyond its structural characteristics, β-caryophyllene displays a distinctive pharmacodynamic profile that differentiates it from other plant-derived cannabinoids [10]. It acts as a selective agonist at cannabinoid receptor type 2 (CB2), which is primarily expressed in immune cells and peripheral tissues, without significant activation of cannabinoid receptor type 1 (CB1) and, consequently, without psychoactive effects [11,12]. Through CB2 engagement, β-caryophyllene modulates key molecular pathways involved in inflammatory and metabolic regulation, including inhibition of nuclear factor kappa B (NF-κB), suppression of pro-inflammatory cytokine production, and interaction with peroxisome proliferator-activated receptors (PPARs), particularly PPAR-α and PPAR-γ [13,14]. These mechanisms provide a mechanistic basis for its reported anti-inflammatory, analgesic, antioxidant, metabolic, neuroprotective, gastroprotective, and immunomodulatory activities observed in preclinical models [15,16,17].

Despite this favorable pharmacological profile and evidence of low acute and subacute toxicity following oral administration, the pharmaceutical translation of β-caryophyllene remains limited by formulation-related challenges [18]. Its high lipophilicity and negligible aqueous solubility hinder incorporation into conventional dosage forms and compromise bioavailability [19]. These limitations are particularly critical in the context of parenteral administration, which requires stable, biocompatible systems that ensure predictable in vivo behavior and minimize local tissue reactions [20]. To date, injectable formulations containing β-caryophyllene are scarce, and systematic investigations addressing its local and systemic safety following parenteral exposure remain limited, representing a relevant gap in the current state of the art [21].

Nanotechnology has emerged as a rational and established strategy to overcome the physicochemical and biopharmaceutical limitations associated with lipophilic natural compounds [22,23]. Nanostructured drug delivery systems enable precise control over particle size, surface properties, and release kinetics, thereby improving solubility, stability, biodistribution, and interaction with biological tissues [24,25]. Among these systems, nanoemulsions have gained considerable prominence due to their relative simplicity of production, kinetic stability, and favorable colloidal characteristics [26]. Typically formulated as oil-in-water dispersions stabilized by surfactants and presenting droplet sizes below 200 nm, nanoemulsions are particularly suitable for the delivery of lipophilic compounds and benefit from a long-standing history of safe use in parenteral nutrition and injectable drug delivery [27].

Intramuscular administration is frequently employed for drugs requiring sustained release or when oral bioavailability is inadequate; however, this route necessitates careful evaluation of tissue compatibility [28]. Inappropriate formulations may induce local inflammation, fibrosis, or muscle damage, highlighting the importance of comprehensive preclinical toxicological assessment [29,30]. In this context, the development of an injectable nanoemulsion represents a promising approach to enable the parenteral delivery of β-caryophyllene. Therefore, the present study aimed to develop and physicochemically characterize an injectable nanoemulsion containing β-caryophyllene and to evaluate its preclinical safety following subacute intramuscular administration in male Wistar rats, with emphasis on clinical observations, hematological and biochemical parameters, and histopathological assessment of muscle, liver, and kidney tissues, in accordance with internationally accepted guidelines [31].

## 2. Results

### 2.1. Formulation Development and Physicochemical Characterization

Five β-caryophyllene nanoemulsions (NBCP1–NBCP5) were prepared using a low-energy emulsification method, maintaining constant amounts of β-caryophyllene and aqueous phase while varying the surfactant ratios to obtain HLB values ranging from 5 to 9. The composition of each formulation is summarized in Table 1.

**Figure 1:**
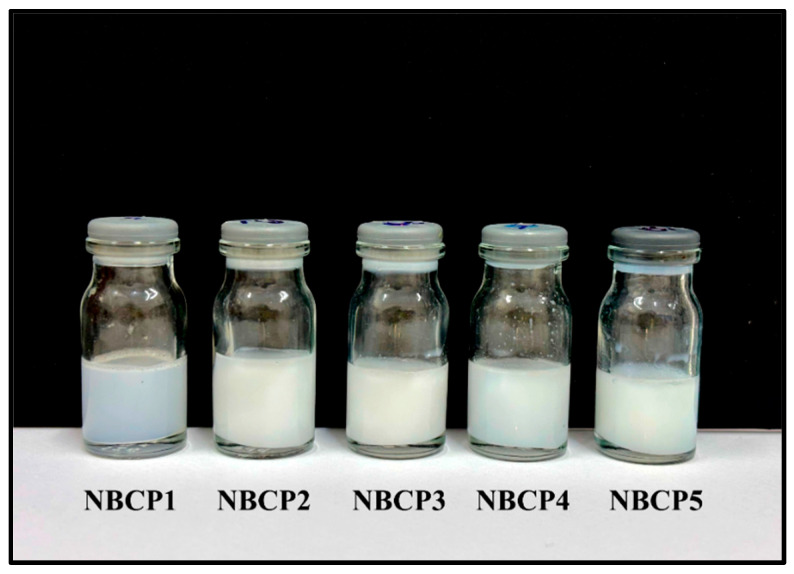
Macroscopic appearance of β-caryophyllene nanoemulsions on the first day of stability evaluation.

On the first day of evaluation, all formulations exhibited a homogeneous and milky appearance, with no visible phase separation (Figure 1).

Droplet size, polydispersity index (PDI), and zeta potential were determined immediately after preparation (day 0) and after 28 days of storage at room temperature. On day 28, mean droplet sizes ranged from 102.39 ± 5.71 nm to 219.60 ± 5.66 nm, PDI values ranged from 0.37 ± 0.01 to 0.46 ± 0.01, and zeta potential values ranged from −27.5 ± 0.6 mV to −13.10 ± 0.36 mV (Table 2).

**Figure 2:**
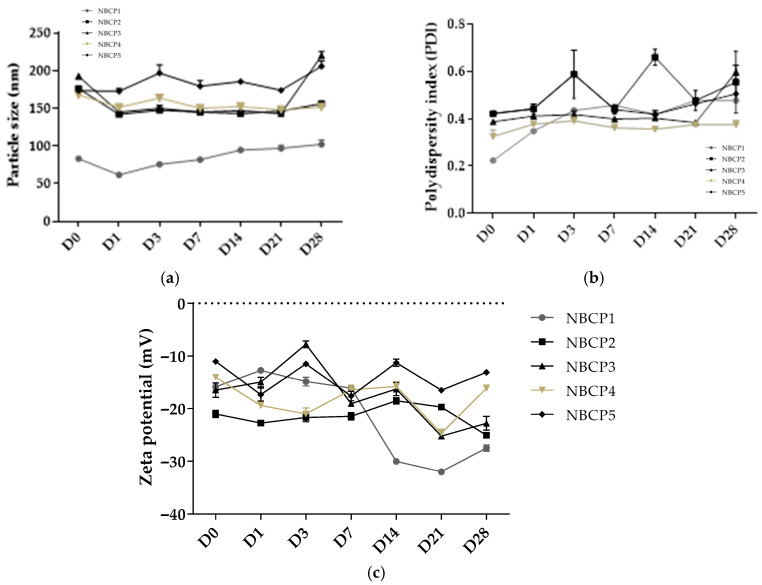
Physicochemical characterization of β-caryophyllene nanoemulsions over 28 days: (**a**) particle size distribution, (**b**) polydispersity index (PDI), and (**c**) zeta potential (Z-average), determined by dynamic light scattering (Zetasizer ZS, Malvern, UK).

The evolution of droplet size over the 28-day period for each formulation is presented in Table 3 and Figure 2.

**Figure 3:**
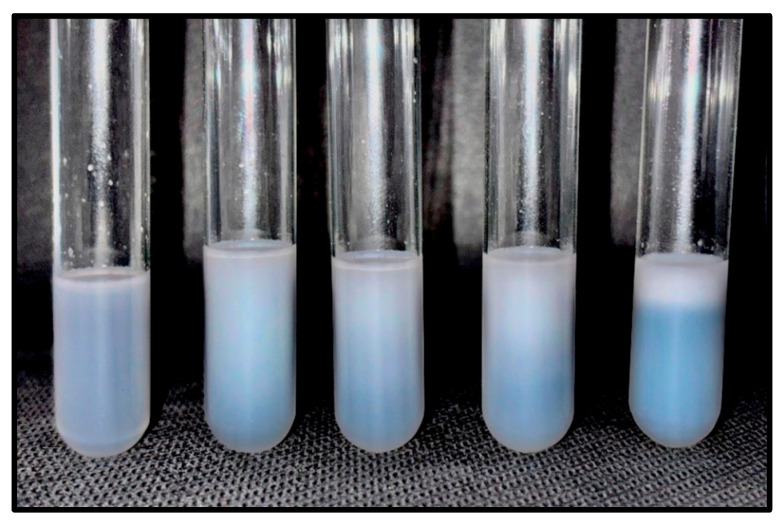
Macroscopic appearance of β-caryophyllene nanoemulsions after centrifugation stress, showing phase separation in NBCP2, NBCP3, NBCP4, and NBCP5.

At day 28, all formulations were subjected to centrifugation and thermal stress tests. After centrifugation, NBCP2, NBCP3, NBCP4, and NBCP5 showed visible signs of phase separation, whereas NBCP1 showed no visual changes. Under thermal stress conditions, all formulations were classified as stable. The results of these tests are summarized in Table 4, and representative images after centrifugation are shown in Figure 3.

Based on the centrifugation test outcome, NBCP1 was selected for subsequent analytical and in vivo studies.

**Figure 4:**
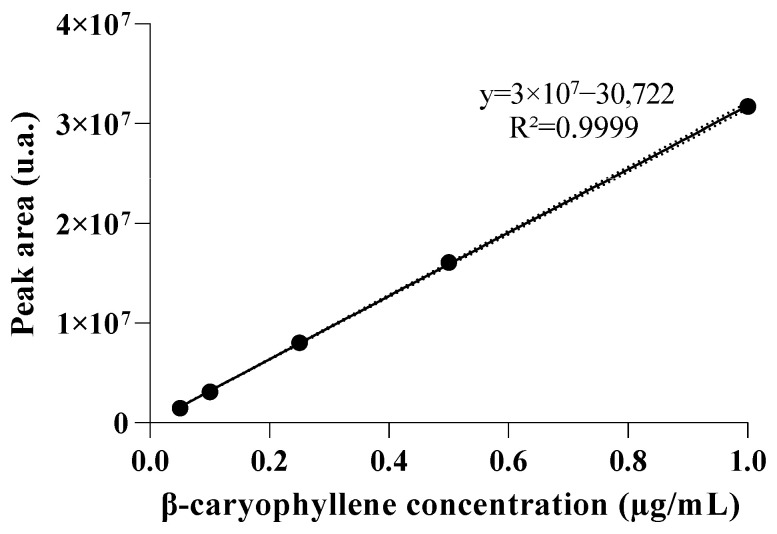
Calibration curve of β-caryophyllene obtained by gas chromatography (GC–MS), showing the relationship between concentration and chromatographic peak area (*y* = 3 × 10^7^*x* − 30,722; R^2^ = 0.9999).

The concentration of β-caryophyllene in NBCP1 was determined using a calibration curve obtained by gas chromatography. The calibration curve showed linearity over the tested range and was described by the equation y = 3 × 10^7^x − 30,722, with a coefficient of determination (R^2^) of 0.9999 (Figure 4).

Using this calibration, the concentration of free β-caryophyllene in the aqueous fraction of NBCP1 was determined to be 0.283 mg/mL. Considering the supplier-reported purity (≥80%) of the compound used during formulation, the purity-corrected theoretical total concentration of β-caryophyllene corresponded to approximately 8.0 mg/mL.

The free fraction, therefore, accounted for 3.54% of the total content, yielding an encapsulation efficiency (EE%) of 96.46%. These findings indicate that the majority of β-caryophyllene remained associated with the oil droplets of the nanoemulsion, demonstrating high loading capacity of the developed system.

**Figure 5:**
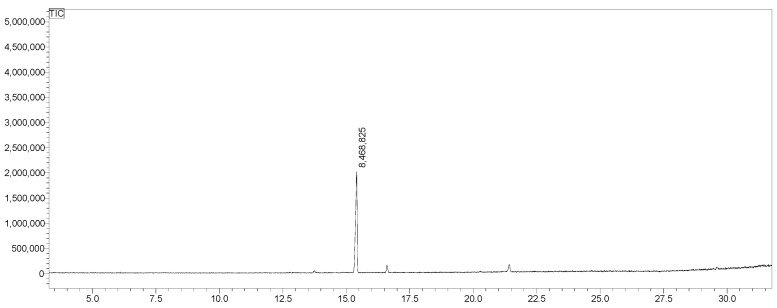
GC–MS chromatogram of β-caryophyllene detected in the free fraction of the NBCP1 nanoemulsion.

**Figure 6:**
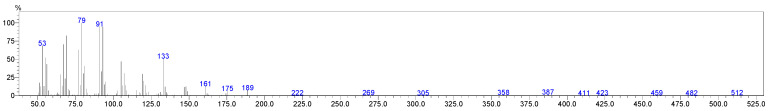
GC–MS chromatogram of β-caryophyllene detected in the free fraction of the NBCP1 nanoemulsion. Mass spectrum of the peak corresponding to β-caryophyllene identified in the free fraction of the NBCP1 nanoemulsion.

The identity of β-caryophyllene in the free fraction was confirmed by GC–MS analysis; the chromatogram showed a distinct peak with a retention time of approximately 15 min (Figure 5), and the corresponding mass spectrum exhibited characteristic ions at m/z 93, 107, 119, and 133 (Figure 6).

### 2.2. In Vivo Safety Assessment

Clinical observations were conducted daily to assess potential signs of toxicity, including behavioral changes, motor function, and autonomic effects. No clinical or behavioral abnormalities were observed during the 14-day observation period in any experimental group following intramuscular administration of NBCP1. All evaluated parameters in the Hippocratic screening, including reflexes, motor activity, autonomic responses, and mortality, remained unchanged across groups (Table 5).

**Figure 7:**
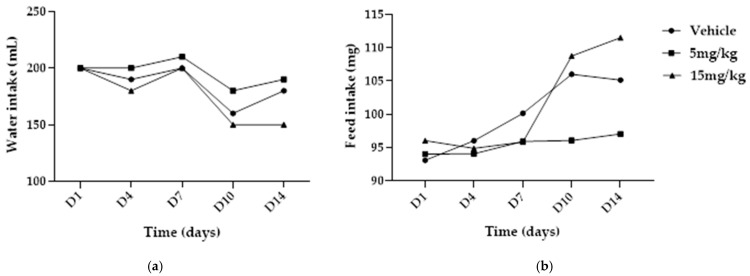
Effect of subacute intramuscular treatment with NBCP (5 and 15 mg/kg) on water and feed intake in male Wistar rats (*n* = 5). (**a**) Time course of water intake. (**b**) Time course of feed intake. Data are presented as descriptive group consumption over the 14-day treatment period.

Water and feed intake were monitored per cage throughout the experimental period. The temporal profiles of water and feed consumption over 14 days are presented in Figure 7.

**Figure 8:**
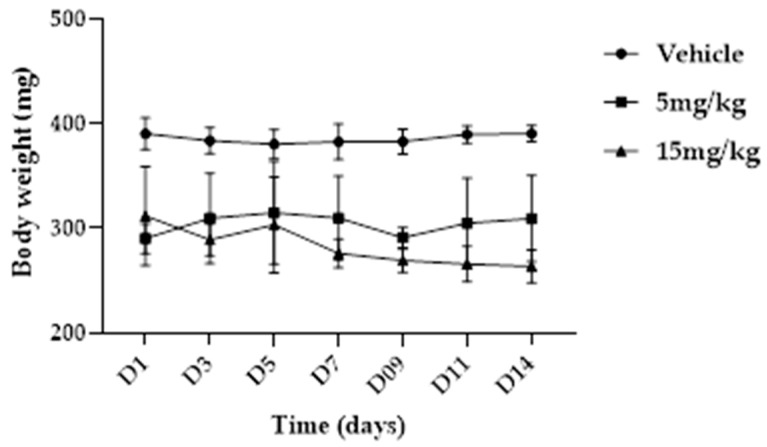
Effect of subacute intramuscular treatment with NBCP (5 and 15 mg/kg) on body weight of male Wistar rats over 14 days (*n* = 5). Data are presented as mean ± SD. Differences between groups were analyzed by one-way analysis of variance (ANOVA) followed by Tukey’s post hoc test. *p* < 0.05 versus control group.

Body weight was recorded during the study, and the evolution of mean body weight over time is shown in Figure 8. No treatment-related differences in body weight progression were detected between groups during the experimental period.

**Figure 9:**
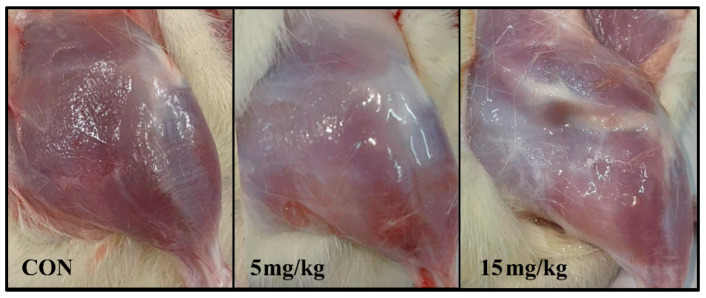
Macroscopic appearance of the injected muscle after intramuscular administration of vehicle (CON—control group) and NBCP at doses of 5 and 15 mg/kg.

At necropsy, organ weights were determined for spleen, pancreas, heart, liver, kidneys, and lungs. No differences were observed between treated and control groups for spleen, pancreas, heart, kidneys, or lungs. Liver weight was higher in the 15 mg/kg group compared with the control group (*p* < 0.05), while no difference was observed between the 5 mg/kg and control groups (Table 6). The macroscopic appearance of the injected muscle is shown in Figure 9.

Hematological parameters, including erythrocyte indices, leukocyte counts and differential, and platelet counts, are presented in Table 7. No statistically significant differences were observed between treated and control groups for any hematological parameter.

**Figure 10:**
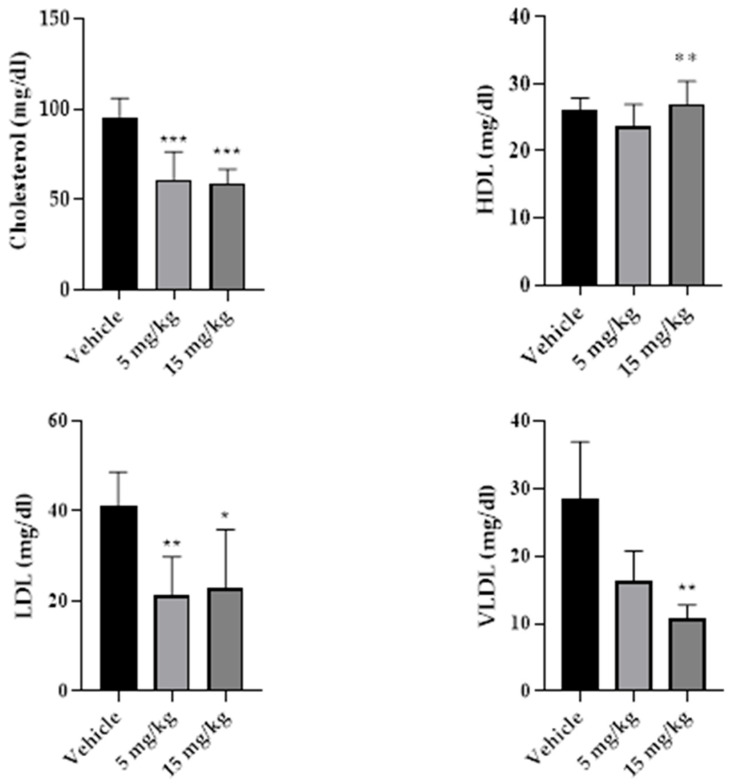
Serum lipid profile parameters were measured after subacute intramuscular treatment with β-caryophyllene nanoemulsion (NBCP) at doses of 5 and 15 mg/kg over a 14-day treatment period. Total cholesterol, HDL cholesterol, LDL cholesterol, and VLDL cholesterol levels in male Wistar rats treated with vehicle, NBCP 5 mg/kg, or NBCP 15 mg/kg for 14 days. Data are presented as mean ± SD (*n* = 5). Statistical differences were assessed by one-way analysis of variance (ANOVA) followed by Tukey’s post hoc test. * *p* < 0.05, ** *p* < 0.01, *** *p* < 0.001 versus vehicle group.

**Figure 11:**
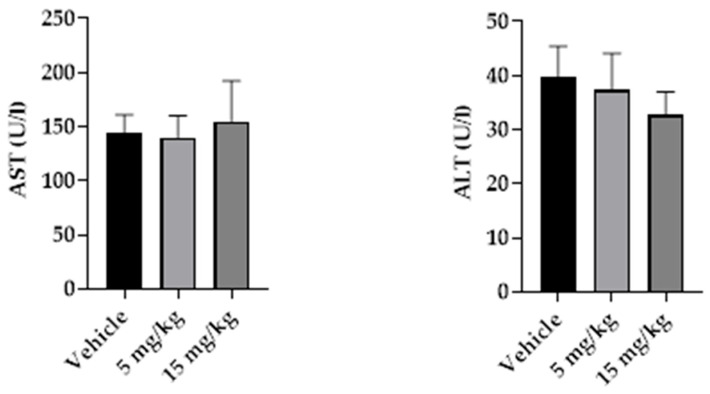
Serum aminotransferase levels after subchronic intramuscular treatment with β-caryophyllene nanoemulsion (NBCP). Alanine aminotransferase (ALT) and aspartate aminotransferase (AST) levels in male Wistar rats treated with vehicle, NBCP 5 mg/kg, or NBCP 15 mg/kg for 14 days. Data are presented as mean ± SD (*n* = 5). Statistical analysis was performed by one-way analysis of variance (ANOVA).

Serum biochemical parameters are summarized in Table 8. Total cholesterol and LDL levels were lower in treated groups, while HDL levels were higher in the 15 mg/kg group. Triglyceride and VLDL levels were reduced in the 15 mg/kg group. Serum creatinine levels were lower in the 15 mg/kg group. AST, ALT, urea, and glucose values are also reported in Table 8 and Figure 10 and Figure 11.

**Figure 12:**
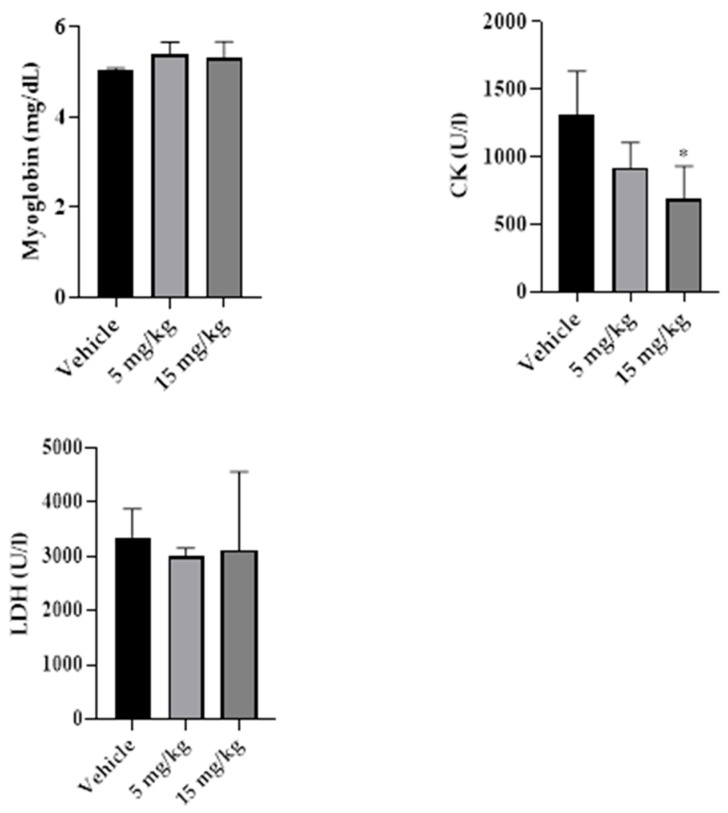
Serum muscle injury markers after subchronic intramuscular treatment with β-caryophyllene nanoemulsion (NBCP). Myoglobin, creatine kinase (CK), and lactate dehydrogenase (LDH) levels in male Wistar rats treated with vehicle, NBCP 5 mg/kg, or NBCP 15 mg/kg for 14 days. Data are presented as mean ± SD (*n* = 5). Statistical differences were assessed by one-way analysis of variance (ANOVA) followed by Tukey’s post hoc test. * *p* < 0.05 versus vehicle group.

Markers of muscle injury, including creatine kinase (CK), lactate dehydrogenase (LDH), and myoglobin, are summarized in Table 9. CK levels were lower in the 15 mg/kg group than in the control group (*p* < 0.05), whereas LDH and myoglobin levels did not differ between groups. The corresponding graphical data are shown in Figure 12.

### 2.3. Histopathological Analysis

#### 2.3.1. Liver

**Figure 13:**
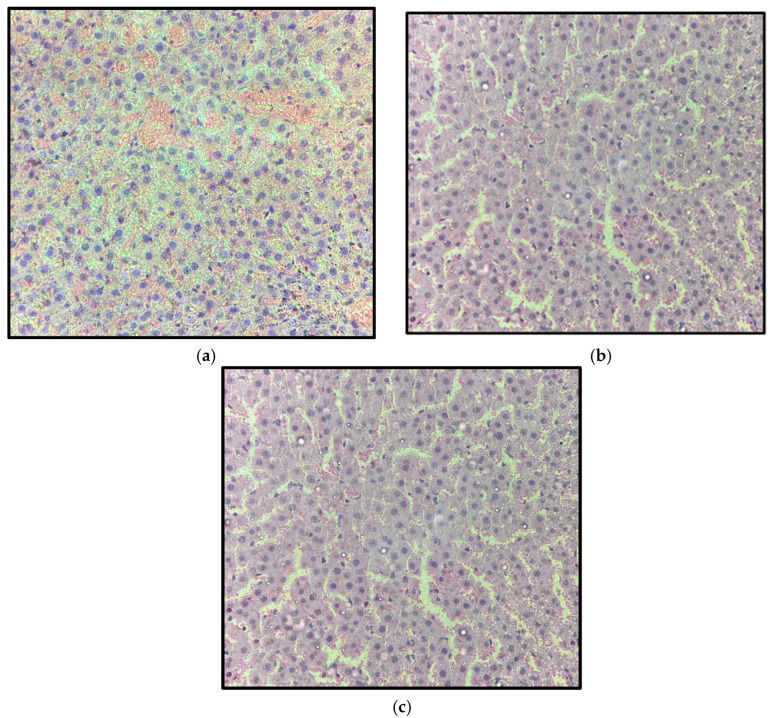
Representative liver sections from control (**a**), 5 mg/kg (**b**), and 15 mg/kg (**c**) groups after 14 days of treatment with β-caryophyllene nanoemulsion (NBCP). Preserved hepatic architecture is observed in all groups, with organized hepatocyte cords, centrally located nuclei, and well-defined sinusoids, without evidence of necrosis, inflammatory infiltrates, or marked degenerative changes (hematoxylin and eosin, H&E; objective 40×; total magnification 400×).

Representative liver sections from the control, 5 mg/kg, and 15 mg/kg groups, collected after 14 days of treatment, are shown in Figure 13. Histological examination revealed preserved hepatic architecture in all groups, characterized by organized hepatocyte cords, centrally located nuclei, and well-defined sinusoids. No evidence of hepatocellular necrosis, inflammatory infiltrates, vascular congestion, or marked degenerative changes was observed in any of the evaluated groups.

#### 2.3.2. Kidney

**Figure 14:**
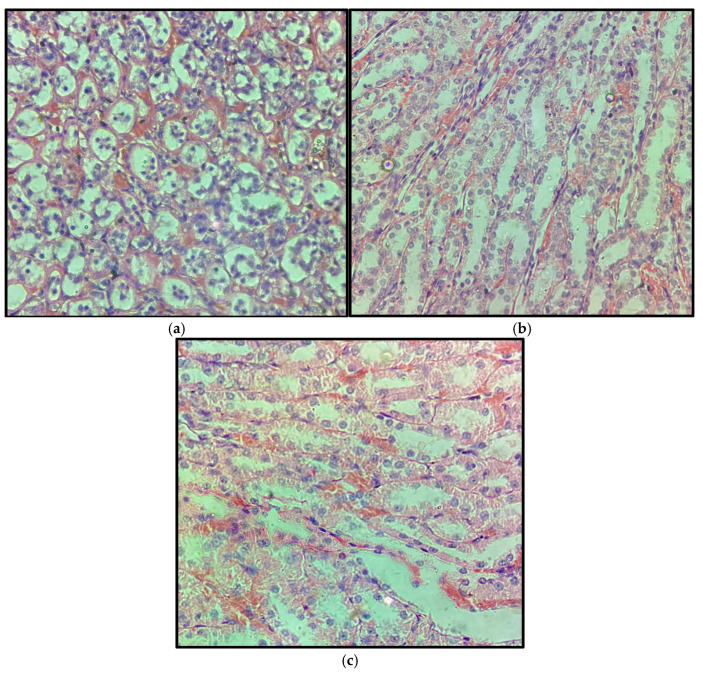
Representative kidney sections from control (**a**), 5 mg/kg (**b**), and 15 mg/kg (**c**) Wistar rats after 14 days of treatment with β-caryophyllene nanoemulsion (NBCP). Preserved renal architecture is observed in all groups, with intact renal tubules and glomeruli, without evidence of tubular necrosis, epithelial degeneration, inflammatory infiltrates, or significant glomerular alterations (hematoxylin and eosin, H&E; objective 40×; total magnification 400×).

Representative kidney sections from the control, 5 mg/kg, and 15 mg/kg groups, collected after 14 days of treatment, are shown in Figure 14. Renal histology revealed preserved cortical and medullary architecture in all groups, with intact glomeruli and well-defined Bowman’s capsules. Renal tubules exhibited normal epithelial lining, without evidence of tubular degeneration, interstitial inflammation, glomerular alterations, or vascular abnormalities.

#### 2.3.3. Injected Muscle

**Figure 15:**
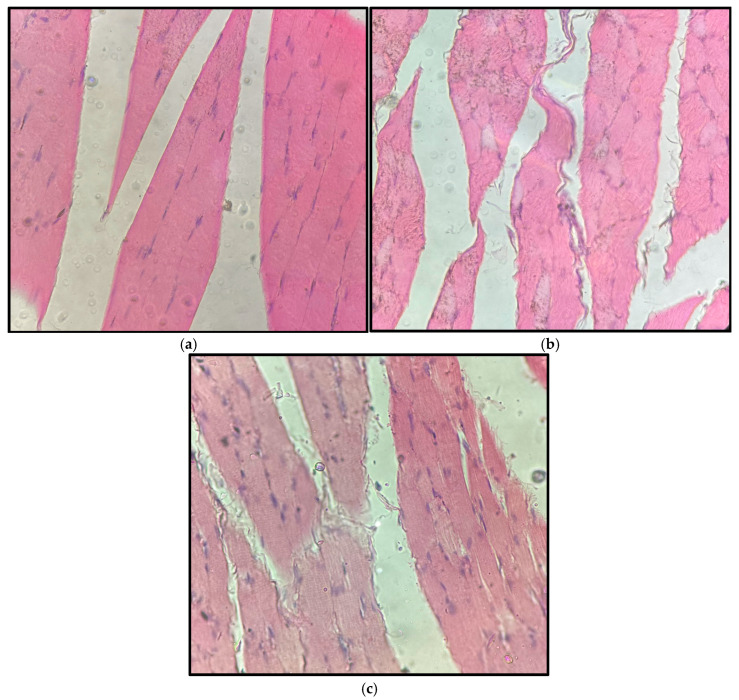
Representative skeletal muscle sections from control (**a**), (**b**), 5 mg/kg, and (**c**) 15 mg/kg groups after 14 days of treatment with β-caryophyllene nanoemulsion (NBCP). Striated muscle fibers with intact sarcolemma, preserved peripheral nuclei, and uniform fascicular arrangement are observed in all groups. No evidence of myofiber degeneration, necrosis, inflammatory infiltrates, or significant interstitial edema was identified (hematoxylin and eosin, H&E; objective 40×; total magnification 400×).

Representative skeletal muscle sections from the injection site of control, 5 mg/kg, and 15 mg/kg groups collected after 14 days of treatment are shown in Figure 15. Histological evaluation demonstrated preserved muscle fiber architecture in all groups, with polygonal fibers, peripheral nuclei, and intact endomysial organization. No evidence of myofiber necrosis, inflammatory infiltrates, hemorrhage, or fibrotic changes was observed at the injection site.

## 3. Discussion

### 3.1. Formulation Design, HLB-Driven Optimization, and Physicochemical Stability

The selection of NBCP1 as the lead formulation indicates that matching the surfactant blend to the required HLB of β-caryophyllene was a key step in obtaining a kinetically stable oil-in-water nanoemulsion. Proper adjustment of the surfactant composition likely improved interfacial coverage and reduced destabilization mechanisms commonly associated with nanoemulsions, such as droplet coalescence and gravitational separation. This interpretation is consistent with previous reports indicating that reduced droplet size and effective interfacial stabilization are closely related to improved colloidal stability [32].

Statistical analysis of droplet diameter and polydispersity index (PDI) demonstrated significant effects of formulation composition and storage time, indicating that the nanoemulsion structure evolved during storage. Some formulations, particularly NBCP1 and NBCP3, exhibited significant temporal variations in droplet diameter and PDI, suggesting progressive interfacial rearrangement within the dispersed system. These differences are likely associated with variations in surfactant composition and HLB values, which influence interfacial tension, droplet stabilization efficiency, and resistance to droplet growth. Nevertheless, all formulations-maintained droplet diameters within the nanometric range throughout the study, indicating preservation of the colloidal nanoemulsion structure.

The non-monotonic behavior observed in droplet size and zeta potential during early storage is consistent with the dynamic nature of nanoemulsions as metastable colloidal systems. Shortly after preparation, interfacial rearrangement of surfactant molecules and transient droplet–droplet interactions may temporarily alter hydrodynamic diameter and surface charge. Subsequent redistribution of surfactants at the oil–water interface can lead to partial interfacial re-equilibration and stabilization of the system. Therefore, moderate oscillations in particle size during early storage should not necessarily be interpreted as irreversible instability, but rather as part of the natural relaxation dynamics of nanoemulsion systems [33].

Zeta potential values became progressively more negative during storage, suggesting modification of the interfacial environment and increased electrostatic repulsion between droplets. Although the absolute values remained below the classical ±30 mV threshold typically associated with purely electrostatic stabilization, the presence of nonionic surfactants indicates that steric stabilization mechanisms also contributed to maintaining the dispersed system. Therefore, the combined interpretation of droplet diameter, PDI, and zeta potential provides a more comprehensive understanding of the stability behavior of the developed nanoemulsions.

Importantly, the selection of NBCP1 for subsequent in vivo evaluation was not based exclusively on statistical differences observed in particle size parameters. Instead, the final decision was based on the overall physicochemical stability profile of the formulations, particularly the macroscopic stability tests. Among the tested systems, NBCP1 was the only formulation that remained stable after centrifugation and thermal stress evaluations, whereas the other formulations showed visible signs of phase separation under accelerated conditions. These findings indicate that NBCP1 exhibited the most robust resistance to destabilization phenomena, such as creaming and coalescence, which are critical parameters for further pharmaceutical development.

Taken together, these results reinforce that low-energy emulsification methods can generate stable nanosystems when formulation variables are carefully optimized. Similar stability ranges have been reported for nanoemulsions containing plant-derived oils, suggesting that rational interfacial design may play a more decisive role than processing intensity alone [34]. Within this framework, NBCP1 demonstrated the most favorable balance between droplet size, interfacial stability, and resistance to accelerated destabilization tests, supporting its selection for subsequent pharmacological and toxicological evaluation.

### 3.2. Encapsulation Performance and Analytical Confirmation

The encapsulation behavior observed for NBCP1 is consistent with expectations for a highly lipophilic sesquiterpene. A limited free fraction in the aqueous phase typically reflects strong solubilization within the oil core combined with an interfacial film that restricts diffusion into the external phase. Chromatographic quantification, supported by GC–MS confirmation, strengthens confidence that the detected signal corresponded specifically to β-caryophyllene rather than co-eluting formulation components. Together, these results indicate that the formulation behaved as intended, retaining the compound predominantly within the dispersed phase.

This aspect is relevant not only for dose delivery but also for local tolerability. Limiting the freely dissolved fraction may reduce immediate exposure peaks at the injection site. Although release kinetics were not assessed in the present study, the analytical profile provides a sound rationale for future investigations focused on release behavior and in vivo exposure.

### 3.3. In Vivo Tolerability: Clinical Observations, Feeding Behavior, and Body Weight

Throughout the subacute protocol, the absence of clinically relevant findings in the Hippocratic screening, together with stable feeding and drinking behavior, suggests that repeated intramuscular administration did not induce overt systemic distress. In repeated-dose toxicology, early behavioral or autonomic disturbances often precede laboratory abnormalities. Therefore, preserved reflex responses and intake patterns are commonly interpreted as supportive evidence of tolerability [35,36,37,38].

Within the framework of the working hypothesis, these endpoints are not merely ancillary. They indicate that neither the excipient system nor the tested dose range was associated with behavioral toxicity or reduced well-being under the applied regimen.

### 3.4. Organ Weights and Interpretation of Isolated Liver Weight Differences

Interpreting isolated organ-weight changes remains a challenge in repeated-dose studies, particularly in the absence of converging biochemical or histological evidence. The liver is especially sensitive to adaptive responses because it is the primary site of xenobiotic metabolism. Accordingly, liver weight differences are best interpreted using a weight-of-evidence approach rather than as isolated toxicity signals. This view is supported by studies emphasizing that organ weight changes become toxicologically relevant primarily when they coincide with functional or morphological injury [39,40,41].

The literature on terpene-containing preparations provides precedent for adaptive hepatic changes without hepatocellular injury. Several reports describe liver weight variations accompanied by preserved transaminase levels and unremarkable histology, frequently interpreted as metabolic adaptation rather than toxicity [42,43,44]. Within this interpretive framework, the present findings remain compatible with an adaptive response, particularly because histopathology and liver enzyme profiles did not indicate injury. This integrated interpretation supports the hypothesis that the nanoemulsion platform can be administered repeatedly without inducing organ-damaging systemic toxicity at the tested doses.

### 3.5. Hematological Safety in the Context of Repeated-Dose Exposure

Hematological stability provides an additional dimension of safety, as blood cell lineages respond rapidly to systemic inflammation, immune activation, or bone marrow suppression. Overall, erythrocyte indices and total leukocyte counts remained within physiological ranges, indicating preserved hematopoietic function following repeated intramuscular administration.

A modest but statistically significant increase in the proportion of segmented neutrophils was observed in the treated groups. However, this change did not follow a clear dose-dependent pattern and occurred in the absence of alterations in total leukocyte counts, other leukocyte subpopulations, or clinical signs of systemic inflammation. Importantly, all observed values remained within reference ranges reported for the species and strain, supporting a physiological rather than adverse interpretation.

Such variations are commonly reported in repeated-dose studies and may reflect normal biological variability related to factors such as strain, sex, handling, or transient adaptive immune responses rather than toxicologically relevant effects. In the absence of corroborating biochemical, histopathological, or clinical findings, isolated changes in differential leukocyte counts are generally not considered indicative of hematopoietic toxicity [45].

When evaluated alongside the preserved clinical profile, the hematological findings strengthen the conclusion that the formulation did not introduce systemic liabilities that would compromise its suitability as a parenteral carrier.

### 3.6. Serum Biochemistry: Hepatic and Renal Function and the Metabolic Signature of β-Caryophyllene

The biochemical profile observed after repeated dosing is consistent with preserved hepatic and renal function. The absence of transaminase elevation supports the interpretation that the formulation did not induce hepatocellular damage under the tested conditions. This finding is coherent with mechanistic literature describing hepatoprotective pathways associated with β-caryophyllene, often linked to CB2 activation and antioxidant signaling, although these pathways were not directly investigated here [46]. Similarly, preserved renal markers align with the absence of histological alterations and support nephrocompatibility for the tested route and duration.

Beyond safety markers, the modulation of the lipid profile observed in the treated groups is noteworthy, as it parallels the pharmacological effects previously reported for β-caryophyllene. Prior studies have described hypolipidemic activity potentially mediated by modulation of hepatic lipid metabolism and inflammatory-metabolic pathways [47]. The increase in HDL levels, particularly at the higher dose, differs from some earlier observations [48]. One plausible explanation is that formulation-driven differences in solubilization or exposure kinetics may contribute to this effect, although this remains speculative in the absence of pharmacokinetic data.

Importantly, interpreting these metabolic shifts within the hypothesis framework suggests an added value of the platform. It may preserve or possibly enhance biologically relevant activities of β-caryophyllene while maintaining a safety profile compatible with repeated intramuscular dosing.

### 3.7. Local Tolerability and Muscle Safety

For intramuscular formulations, local tolerability is a critical determinant of feasibility. The absence of increased muscle injury biomarkers, together with preserved muscle architecture on histological analysis, indicates that repeated injections were well tolerated at the tissue level. This contrasts with reports describing local reactogenicity associated with certain injectable excipients, including inflammatory and fibrotic responses observed with some surfactant-based vehicles [49]. In this context, the present profile suggests that the NBCP formulation did not reproduce these local liabilities.

The reduction in creatine kinase observed at the higher dose is also consistent with an absence of muscle injury. Although this finding may reflect variability, it is compatible with an anti-inflammatory context previously described for β-caryophyllene-containing preparations. Prior studies have reported reductions in muscle discomfort and injury-related biomarkers following muscle stress, attributed to modulation of inflammatory and nociceptive pathways [50,51]. Preclinical evidence further supports suppression of pro-inflammatory mediators by β-caryophyllene [52]. While the present study was not designed to assess muscle-protective efficacy, the combined biomarker and histological profiles are consistent with local biocompatibility and do not suggest myotoxicity.

### 3.8. Histopathological Congruence as a Weight-of-Evidence Endpoint

Histopathological analysis provides a decisive layer of interpretation by confirming whether biochemical stability reflects preserved tissue integrity. The preserved hepatic architecture observed here aligns with previous subchronic exposure studies of β-caryophyllene reporting the absence of relevant liver pathology [53]. Similarly, renal histology corroborates the biochemical findings and is consistent with literature describing low nephrotoxic potential and, in some contexts, nephroprotective effects mediated by anti-inflammatory and antioxidant mechanisms [54,55].

Some studies have described mild renal alterations following prolonged exposure to terpene-rich mixtures at high doses [56,57]. The absence of comparable findings in the present study suggests that the formulation, dose, and intramuscular route may offer a favorable safety margin.

Overall, convergence among clinical observations, laboratory markers, and histological findings strengthens the safety assessment more than any individual endpoint. This integrated profile is particularly relevant for parenteral development, where both systemic tolerability and local tissue compatibility are required.

### 3.9. Broader Implications for Injectable Nanotechnology and Natural Product Delivery

This study addresses a practical challenge in pharmaceutical nanotechnology, namely the parenteral delivery of highly lipophilic natural products without relying on harsh solvents or reactogenic vehicles. By combining a low-energy manufacturing approach with HLB-guided surfactant optimization, the work outlines a development pathway that is methodologically accessible and potentially scalable. The in vivo profile further suggests that such systems can be designed for repeated intramuscular administration without evident systemic or local toxicity signals in standard toxicological endpoints.

In the broader context of β-caryophyllene research, a stable injectable nanoemulsion may expand experimental possibilities beyond oral administration. It may also enable controlled-exposure studies in models in which route, bioavailability, and tissue distribution are critical variables.

### 3.10. Limitations and Future Directions

Several next steps logically follow from these findings. Pharmacokinetic studies are needed to quantify systemic exposure, tissue distribution, and the relationship between formulation retention and in vivo bioavailability after intramuscular dosing. Efficacy should then be evaluated in disease-relevant models aligned with β-caryophyllene pharmacology, particularly those related to inflammation and pain, to determine whether the nanoemulsion format influences onset, magnitude, or duration of effect.

Longer-term safety assessments, including chronic dosing and recovery periods, would further strengthen the toxicological profile and help distinguish adaptive from adverse changes, particularly regarding liver weight findings. Finally, the same HLB-driven strategy could be applied to other lipophilic terpenes or natural products, allowing evaluation of the platform’s broader applicability.

## 4. Materials and Methods

### 4.1. Materials

β-caryophyllene (≥80% purity; lot SHBM3771) was obtained from Sigma-Aldrich Inc. (St. Louis, MO, USA). All mass-balance calculations were performed considering the supplier-reported purity of the compound. Sorbitan monooleate (Span 80) and polysorbate 80 (Tween 80) were purchased from Êxodo Científica Co. (Sumaré, SP, Brazil) and used as non-ionic surfactants. All solvents used for chromatographic analyses were of analytical or chromatographic grade. Deionized water was used in all experiments.

### 4.2. Development and Optimization of β-Caryophyllene Nanoemulsions

Nanoemulsions were prepared using a low-energy emulsification method based on phase inversion and determination of the required hydrophilic–lipophilic balance (HLBr), with adaptations from previously described protocols [32,58,59,60]. For each nanoemulsion system (NBCP1–NBCP5), five independent batches were prepared, each with a final volume of approximately 20 mL. Therefore, a total volume of approximately 100 mL per formulation was available for physicochemical characterization and stability assessment. The oil phase, containing β-caryophyllene and surfactants, was magnetically stirred at 800 rpm for 30 min, followed by dropwise addition of the aqueous phase at 0.5 mL·min^−1^ under continuous stirring for an additional 30 min. The resulting nanoemulsions were sterilized by autoclaving.

The HLBr of β-caryophyllene was determined using mixtures of polysorbate 80 (HLB 15.0) and sorbitan monooleate (HLB 4.3) to obtain formulations with HLB values ranging from 5 to 12, calculated as:

HLBr = (HLB a × mA + HLB b × mB)/(mA + mB)
(1)

where HLBr is the required hydrophilic–lipophilic balance of the surfactant mixture; “HLB a” and “HLB b” correspond to the HLB values of surfactants A and B; and “mA” and “mB” represent the mass (g) of each surfactant in the formulation. The equation represents the weighted average HLB of the surfactant blend based on their respective mass contributions. In this study, surfactant A corresponds to Span 80 (HLB = 4.3), and surfactant B corresponds to Tween 80 (HLB = 15.0), according to the manufacturers’ specifications.

Five formulations (NBCP1–NBCP5) were obtained. NBCP1 was selected for subsequent in vivo studies based on predefined selection criteria, including mean droplet size, polydispersity index, zeta potential, and physicochemical parameters obtained during physicochemical characterization.

### 4.3. Physicochemical Characterization

Nanoemulsions containing 1% (*w*/*w*) β-caryophyllene were prepared using an oil-to-surfactant ratio of 1:1 (*w*/*w*) and stored at 25 °C. Each nanoemulsion was prepared with approximately 20 g of total formulation containing 0.2 g of β-caryophyllene raw material (≥80% purity), corresponding to 0.16 g of pure compound and a final theoretical concentration of approximately 0.8% (*w*/*w*) of β-caryophyllene in the formulation.

Physical stability was assessed on days 0, 1, 3, 7, 14, and 28 by macroscopic evaluation; the period represents a short-term stability evaluation commonly used during formulation screening and optimization, rather than a long-term stability study [61]. Droplet size, polydispersity index (PDI), and zeta potential were determined by dynamic light scattering, as previously described [62,63].

Particle size (Z-average diameter) and polydispersity index (PDI) were determined by dynamic light scattering (DLS) using a Zetasizer ZS instrument (Malvern Panalytical, UK). Measurements were performed at 25 °C. Prior to analysis, samples were diluted in ultrapure water at an appropriate ratio to avoid multiple scattering effects. Measurements were conducted at a backscattering angle of 173°.

For each sample and time point, three technical replicate measurements were performed, each consisting of at least 10 runs, and results were expressed as mean ± standard deviation (SD). Data were processed using the instrument software to obtain Z-average diameter and PDI values. Zeta potential was measured using the same Zetasizer ZS instrument equipped with electrophoretic light scattering capability. Samples were diluted under the same conditions used for DLS measurements and analyzed at 25 °C.

Zeta potential values were calculated according to the Smoluchowski equation as implemented in the manufacturer’s software. Three technical replicate measurements were performed for each sample and time point.

Encapsulation efficiency (EE%) was determined indirectly by quantifying non-encapsulated β-caryophyllene using gas chromatography–mass spectrometry (GC–MS) [64]. Nanoemulsion samples were ultracentrifuged (20,000–30,000× *g*, 30–60 min, 25 °C), and the supernatant containing free β-caryophyllene was extracted with n-hexane. Total β-caryophyllene was quantified after complete droplet disruption. EE% was calculated as:

EE% = [(BCP total − BCP free)/BCP total] × 100
(2)

where “BCP total” corresponds to the theoretical amount of pure β-caryophyllene added to the formulation, adjusted according to the supplier-reported purity (≥80%), and “BCP free” represents the non-encapsulated fraction quantified by GC analysis.

GC–MS analyses were performed using a DB-5MS capillary column (30 m × 0.25 mm × 0.25 µm). The oven temperature was programmed from 50 °C to 280 °C at 10 °C·min^−1^. Helium was used as the carrier gas (1 mL·min^−1^). Injections were performed in splitless mode (1 µL) with electron-impact ionization at 70 eV. Quantification was carried out using an external calibration curve (0.5–50 µg·mL^−1^).

### 4.4. Statistical Analysis of Physicochemical Parameters

Droplet size (Z-average), polydispersity index (PDI), and zeta potential were analyzed using two-way ANOVA (formulation × time), followed by Tukey’s multiple comparisons test. Measurements were performed in triplicate as technical replicates and expressed as mean ± standard deviation (SD). Statistical significance was set at *p* < 0.05. Analyses were performed using GraphPad Prism (version 8.0).

### 4.5. Animals and Ethical Approval

A randomized, controlled, non-clinical study was conducted at the Laboratory of Pharmaceutical Research, Federal University of Amapá (UNIFAP), Brazil. Male Wistar rats (*Rattus norvegicus albinus*), weighing 200–300 g, were obtained from the Multidisciplinary Center for Biological Investigation in Laboratory Animal Science (CEMIB), University of Campinas (UNICAMP, Brazil). Animals were housed under controlled environmental conditions (25 ± 2 °C, 12 h light/dark cycle), with free access to standard rodent chow and water *ad libitum*.

### 4.6. Experimental Design and Treatment Protocol

Subacute intramuscular tolerability and biocompatibility were evaluated over a 14-day experimental period using an exploratory repeated-dose design. The study was conducted in accordance with internationally accepted principles for laboratory animal care and the ethical standards established by the Brazilian National Council for the Control of Animal Experimentation (CONCEA), following approval by the Institutional Animal Ethics Committee (approval number 015/2018) [65].

Animals were randomly allocated into three experimental groups (*n* = 5 per group): vehicle, NBCP 5 mg/kg, and NBCP 15 mg/kg. The sample size was defined based on ethical considerations aligned with the 3Rs principles (Replacement, Reduction, and Refinement) and is consistent with exploratory toxicological screening approaches aimed at detecting biologically relevant systemic and local effects while minimizing animal use. Although full regulatory repeated-dose toxicity studies (e.g., OECD TG 407) [66] may require larger group sizes, the present investigation was designed as a preliminary safety assessment rather than a definitive regulatory toxicity study.

The vehicle group received the formulation vehicle without β-caryophyllene, containing non-ionic surfactants (Span 80 and Tween 80). Treated groups received the β-caryophyllene-loaded nanoemulsion (NBCP) at doses of 5 or 15 mg/kg. All formulations were administered by intramuscular injection into the quadriceps muscle at predefined intervals throughout the experimental period.

The nanoemulsion selected for in vivo evaluation corresponded to the formulation presenting the highest physicochemical stability and the smallest mean droplet size among the developed formulations.

### 4.7. Clinical Observations and Toxicological Assessment

Animals were observed daily for clinical signs of toxicity throughout the study. An observational screening (Hippocratic screening) was performed to assess general behavior, motor activity, reflexes, and central nervous system function.

The following parameters were evaluated: vocal tremor, irritability, response to touch, tail pinch response, contortion, body tone, grip strength, ataxia, auricular reflex, corneal reflex, tremors and/or convulsions, anesthesia, lacrimation, micturition and defecation, piloerection, cyanosis or hyperemia, and mortality. Observations were performed at 1, 2, 4, and 8 h after administration and subsequently on a daily basis during the experimental period.

Body weight was recorded throughout the experimental period. Food intake and water consumption were monitored daily and expressed as group consumption over the 14-day treatment period.

### 4.8. Histopathological, Hematological and Biochemical Analyses

At the end of the experimental period, animals were euthanized with sodium thiopental (45 mg/kg; Cristália Co., São Paulo, Brazil). Approximately 1.5 mL of blood was collected via retro-orbital plexus puncture for hematological and biochemical analyses. Liver, kidneys, and quadriceps muscle were excised and fixed in 10% buffered formalin.

Tissues were processed according to routine histological procedures, sectioned at 4–5 µm thickness, and stained with hematoxylin and eosin (H&E). Histopathological evaluation was performed using optical microscopy.

Serum biochemical parameters were determined using commercial assay kits (Doles^®^, São Paulo, Brazil) and a UV–VIS spectrophotometer (Shimadzu UVmini 1240, Kyoto, Japan). Hematological parameters were analyzed using an automated hematology analyzer (HumaCount Plus, Human GmbH, Berlin, Germany), as previously described [67,68].

### 4.9. Statistical Analysis

Results were expressed as mean ± standard deviation. Statistical analyses were performed using one-way analysis of variance (ANOVA), followed by Tukey’s post hoc test. Differences were considered statistically significant at *p* < 0.05. Analyses were conducted using GraphPad Instat and GraphPad Prism (version 8.0).

## 5. Conclusions

This study investigated an injectable nanoemulsion containing β-caryophyllene, focusing on its physicochemical characteristics and preclinical safety following intramuscular administration. Among the formulations evaluated, NBCP1 was selected based on its droplet size distribution, polydispersity, surface charge, and stability under stress conditions.

Repeated intramuscular administration over 14 days was not associated with clinically relevant systemic or local toxicity. Hematological and biochemical parameters remained within physiological ranges, and histopathological examination of skeletal muscle, liver, and kidney tissues demonstrated preserved architecture without evidence of inflammatory or degenerative alterations. The increase in relative liver weight observed at the higher dose occurred in the absence of biochemical or histological abnormalities and is consistent with an adaptive response rather than hepatotoxicity.

Collectively, these findings indicate that the NBCP1 nanoemulsion is suitable for the parenteral administration of β-caryophyllene under the conditions evaluated. The data help address the current knowledge gap regarding injectable formulations of this sesquiterpene and provide a rationale for further studies focusing on pharmacokinetics, long-term safety, and therapeutic efficacy.

## 6. Patents

A patent application for the formulation investigated in this study has been filed with the Brazilian National Institute of Industrial Property (INPI) under number BR 10 2025 026011 5.

## Figures and Tables

**Table 1:** Composition of β-caryophyllene nanoemulsion formulations.

Nanoemulsion	HLB	Sorbitan Monooleate (g)	Polysorbate 80 (g)	β-Caryophyllene (g)	Water (g)
NBCP1	5	0.013	0.186	0.2	19.6
NBCP2	6	0.031	0.168	0.2	19.6
NBCP3	7	0.050	0.149	0.2	19.6
NBCP4	8	0.069	0.130	0.2	19.6
NBCP5	9	0.182	0.170	0.2	19.6

**Table 2:** Effect of nanoemulsion composition (NBCPs) on particle diameter (nm), polydispersity index (PDI), and zeta potential.

		Day 1			Day 28		
Nanoemulsion	HLB	Diameter (nm)	PDI	Zeta Potential	Diameter (nm)	PDI	Zeta Potential
NBCP1	5	83.26 ± 0.12	0.22 ± 0.00	−15.9 ± 0.7	102.39 ± 5.71 *	0.55 ± 0.13 *	−27.5 ± 0.6 *
NBCP2	6	176.23 ± 2.68	0.42 ± 0.08	−11.0 ± 0.4	156.16 ± 2.12	0.41 ± 0.01	−25.1 ± 0.5 *
NBCP3	7	192.30 ± 1.73	0.38 ± 0.00	−16.50 ± 1.37	219.6 ± 5.66 *	0.59 ± 0.02 *	−22.80 ± 1.3 *
NBCP4	8	167.9 ± 2.50	0.37 ± 0.01	−14.03 ± 0.38	151.6 ± 1.31	0.37 ± 0.01	−16.17 ± 0.65 *
NBCP5	9	173.5 ± 1.92	0.44 ± 0.01	−11.03 ± 0.42	205.73 ± 6.84 *	0.46 ± 0.01	−13.10 ± 0.36 *

Statistical analysis was performed using two-way ANOVA followed by Tukey’s test. * Different letters within the same column indicate statistically significant differences (*p* < 0.05).

**Table 3:** Effect of nanoemulsion composition (NBCPs) on particle diameter (nm) and formulation stability.

Nanoemulsion	Day 0	Day 1	Day 3	Day 7	Day 14	Day 21	Day 28
NBCP1	83.26 ± 0.12	61.70 ± 0.65 *	75.76 ± 0.37 *	81.96 ± 0.37 *	94.67 ± 0.80	96.93 ± 4.21	102.39 ± 5.71
NBCP2	176.23 ± 2.67	141.93 ± 1.59 *	147.67 ± 6.29	145.13 ± 1.81	143.07 ± 2.25	146.30 ± 5.73	156.17 ± 2.12 *
NBCP3	192.30 ± 1.73	144.93 ± 2.36 *	149.93 ± 1.71 *	144.83 ± 2.68 *	146.97 ± 3.06	143.03 ± 3.57	219.60 ± 5.66 *
NBCP4	167.90 ± 2.51	151.23 ± 3.02 *	163.57 ± 0.68 *	151.60 ± 1.31 *	152.10 ± 0.98	148.17 ± 2.06	151.60 ± 1.31
NBCP5	173.50 ± 1.92	172.60 ± 3.92 *	196.80 ± 10.77	179.33 ± 7.51	185.43 ± 2.25	173.87 ± 3.35 *	205.73 ± 0.02 *

Statistical analysis was performed using two-way ANOVA followed by Tukey’s multiple comparison test. * *p* < 0.05 compared with the immediately preceding time point.

**Table 4:** Physicochemical properties of nanoformulations after centrifugation and thermal stress tests.

	NBCP1	NBCP2	NBCP3	NBCP4	NBCP5
Color	White/translucent	White/opaque	White/opaque	White/opaque	White/opaque
pH	5.13 ± 0.02	5.77 ± 0.01	5.86 ± 0.03	5.82 ± 0.01	5.80 ± 0.01
Density (g/cm^3^)	0.996 ± 0.01	0.962 ± 0.02	0.938 ± 0.02	0.97 ± 0.001	0.967 ± 0.02
Centrifugation	Stable	Unstable	Unstable	Unstable	Unstable
Thermal stress	Stable	Stable	Stable	Stable	Stable

**Table 5:** Hippocratic screening parameters were assessed in rats following subacute intramuscular administration of the β-caryophyllene nanoemulsion (NBCP) at doses of 5 and 15 mg/kg over a 14-day observation period.

Parameters	Vehicle	NBCP 5 mg/kg	NBCP 15 mg/kg
Vocal fremitus	Normal	Normal	Normal
Irritability	Normal	Normal	Normal
Touch response	Normal	Normal	Normal
Tail touch response	Normal	Normal	Normal
Contortions	Normal	Normal	Normal
Body tonus	Normal	Normal	Normal
Grip strength	Normal	Normal	Normal
Ataxia	Absent	Absent	Absent
Auricular reflex	Normal	Normal	Normal
Tremors	Absent	Absent	Absent
Hypnosis/anesthesi	Absent	Absent	Absent
Lacrimation	Absent	Absent	Absent
Urination/defecation	Normal	Normal	Normal
Piloerection	Absent	Absent	Absent
Cyanosis/hyperemia	Absent	Absent	Absent
Death	Absent	Absent	Absent

**Table 6:** Effect of subacute intramuscular treatment with NBCP (5, 10, and 15 mg/kg) on organ weights (g) of male rats after 14 days.

Parameter	Control	5 mg/kg	15 mg/kg
Spleen	1.274 ± 0.658	0.542 ± 0.05	0.766 ± 1.161
Pancreas	2.050 ± 1.308	1.696 ± 1.320	1.016 ± 0.532
Heart	1.792 ± 0.249	0.980 ± 0.492	1.39 ± 0.357
Liver	12.384 ± 0.557	8.840 ± 1.080	9.51 ± 0.828 *
Kidneys	1.698 ± 1.515	0.996 ± 0.073	1.144 ± 0.073
Lung	2.390 ± 1.718	1.552 ± 1.391	1.946 ± 0.339

* Results are presented as mean ± SD (*n* = 5). Differences between groups were analyzed by one-way analysis of variance (ANOVA) followed by Tukey’s post hoc test. * *p* < 0.05 versus the control group.

**Table 7:** Effect of subacute intramuscular treatment with NBCP (5 and 15 mg/kg) on hematological parameters of male *Wistar rats* (*n* = 5) after 14 days.

Parameter	Control	5 mg/kg	15 mg/kg
Erythrogram			
Red blood cells (×10^6^/mm^3^)	9.56 ± 0.21	9.41 ± 0.23	9.26 ± 0.15
Hemoglobin (g/dL)	16.68 ± 0.21	16.04 ± 0.25	16.06 ± 0.35
Hematocrit (%)	50.68 ± 0.84	48.42 ± 1.09	48.40 ± 1.08
MCV (fL)	53.08 ± 0.58	51.48 ± 1.15	52.25 ± 1.12
MCH (pg)	17.44 ± 0.18	17.06 ± 0.40	17.34 ± 0.42
MCHC (g/dL)	32.9 ± 0.16	33.14 ± 0.27	33.18 ± 0.13
RDW (%)	21.140 ± 1.18	20.88 ± 0.82	19.64 ± 1.04
Leukogram			
Leukocytes (×10^3^/mm^3^)	7.85 ± 0.92	8.23 ± 1.65	7.03 ± 0.85
Segmented neutrophils (%)	15.30 ± 1.45	19.74 ± 2.07 *	25.52 ± 10.56 *
Eosinophils (%)	1.38 ± 0.37	1.98 ± 0.50	1.37 ± 0.47
Basophils (%)	11.62 ± 1.77	11.70 ± 1.23	8.44 ± 2.46
Lymphocytes (%)	67.16 ± 2.57	60.36 ± 2.15	59.32 ± 8.67
Monocytes (%)	4.56 ± 0.76	6.22 ± 0.95	5.36 ± 0.50
Platelets			
Platelets (×10^3^/mm^3^)	1063 ± 172	1211 ± 124	1374 ± 121
MVP (fL)	10.88 ± 0.36	10.94 ± 0.56	10.840 ± 0.39

* Mean corpuscular volume (MCV), mean corpuscular hemoglobin (MCH), and mean corpuscular hemoglobin concentration (MCHC). Results are expressed as mean ± SD. Differences between groups were analyzed by one-way analysis of variance (ANOVA) followed by Tukey’s post hoc test. * *p* < 0.05 versus the control group.

**Table 8:** Effect of subacute intramuscular treatment with NBCP (5 and 15 mg/kg) on biochemical parameters of male Wistar rats (*n* = 5) after 14 days.

Parameter	Control	5 mg/kg	15 mg/kg
Glucose (mg/dL)	97.6 ± 23.17	79.3 ± 21.41	71.6 ± 6.81
Total cholesterol (mg/dL)	95.8 ± 8.6	61.2 ± 34.6 *	59.0 ± 36.0 *
HDL cholesterol (mg/dL)	17.8 ± 8.78	23.6 ± 5.8	27.8 ± 9.2 *
LDL cholesterol (mg/dL)	41.22 ± 8.545	21.84 ± 9.57 *	22.84 ± 14.54 *
VLDL cholesterol (mg/dL)	28.52 ± 10.12	16.36 ± 12.16	10.7 ± 7.82 *
Triglycerides (mg/dL)	140.2 ± 31.0	93.8 ± 11.59	53.5 ± 8.98 *
AST (U/L)	144.8 ± 16.27	140.0 ± 20.17	155.20 ± 37.17
ALT (U/L)	39.8 ± 5.63	37.4 ± 6.7	32.8 ± 4.20
Urea (mg/dL)	55 ± 9.46	63.6 ± 9.263	61.0 ± 6.28

High-density lipoprotein cholesterol (HDL); low-density lipoprotein cholesterol (LDL); very-low-density lipoprotein cholesterol (VLDL); aspartate aminotransferase (AST); alanine aminotransferase (ALT). Results are expressed as mean ± SD. Differences between groups were analyzed by one-way analysis of variance (ANOVA) followed by Tukey’s post hoc test. * *p* < 0.05 versus the control group.

**Table 9:** Effect of subacute intramuscular treatment with NBCP (5, 10, and 15 mg/kg) on muscle injury markers in Wistar rats after 14 days.

Parameter	Control	5 mg/kg	15 mg/kg
Myoglobin (mg/dL)	5.040 ± 0.055	5.400 ± 0.055	5.320 ± 0.356
CK (U/L)	1.312 ± 323.833	919.600 ± 184.028	689.0 ± 241.404 *
LDH (U/L)	3357.0 ± 520.448	2998.0 ± 159.830	3116.60 ± 1448.412

* Creatine kinase (CK); lactate dehydrogenase (LDH). Results are expressed as mean ± SD. Differences between groups were analyzed by one-way analysis of variance (ANOVA) followed by Tukey’s post hoc test. * *p* < 0.05 versus control group.

## Data Availability

The data presented in this study are available on request from the corresponding author. The data is not publicly available due to the patent filing process.

## Abbreviations

The following abbreviations are used in this manuscript:

ALT	Alanine aminotransferase
AST	Aspartate aminotransferase
BCP	β-Caryophyllene
CB1	Cannabinoid receptor type 1
CB2	Cannabinoid receptor type 2
CK	Creatine kinase
CON	Control group
EE	Encapsulation efficiency
GC	Gas chromatography
GC-MS	Gas chromatography–mass spectrometry
HDL	High-density lipoprotein
HBL	Hydrophilic–lipophilic balance
IM	Intramuscular
LDH	Lactate dehydrogenase
LDL	Low-density lipoprotein
NBCP	β-Caryophyllene nanoemulsion
NF-κB	Nuclear factor kappa B
PDI	Polydispersity index
PPARs	Peroxisome proliferator-activated receptors
PPAR-α	Peroxisome proliferator-activated receptor alpha
PPAR-γ	Peroxisome proliferator-activated receptor gamma
VLDL	Very-low-density lipoprotein
